# The Effect of Fibrous Reinforcement on the Polycondensation Degree of Slag-Based Alkali Activated Composites

**DOI:** 10.3390/polym13162664

**Published:** 2021-08-10

**Authors:** Isabella Lancellotti, Federica Piccolo, Hoang Nguyen, Mohammad Mastali, Mohammad Alzeer, Mirja Illikainen, Cristina Leonelli

**Affiliations:** 1 Department of Engineering ‘Enzo Ferrari’, University of Modena and Reggio Emilia, Via Vivarelli 10, 41125 Modena, Italy; federica.piccolo@unimore.it (F.P.); cristina.leonelli@unimore.it (C.L.); 2 Fibre and Particle Engineering Research Unit, University of Oulu, Pentti Kaiteran Katu 1, 90014 Oulu, Finland; hoang.nguyen@oulu.fi (H.N.); mohammad.mastali@oulu.fi (M.M.); mohammad.alzeer@oulu.fi (M.A.); mirja.illikainen@oulu.fi (M.I.)

**Keywords:** alkali-activated materials, chemical stability, leaching tests, metallurgical slags, fibers

## Abstract

Alternative cementitious binders, based on industrial side streams, characterized by a low carbon footprint, are profitably proposed to partially replace Portland cement. Among these alternatives, alkali-activated materials have attracted attention as a promising cementitious binder. In this paper, the chemical stability of the matrix, in fiber-reinforced slag-based alkali-activated composites, was studied, in order to assess any possible effect of the presence of the reinforcement on the chemistry of polycondensation. For this purpose, organic fiber, cellulose, and an inorganic fiber, basalt, were chosen, showing a different behavior in the alkaline media that was used to activate the slag fine powders. The novelty of the paper is the study of consolidation by means of chemical measurements, more than from the mechanical point of view. The evaluation of the chemical behavior of the starting slag in NaOH, indeed, was preparatory to the understanding of the consolidation degree in the alkali-activated composites. The reactivity of alkali-activated composites was studied in water (integrity test, normed leaching test, pH and ionic conductivity), and acids (leaching in acetic acid and HCl attack). The presence of fibers does not favor nor hinder the geopolymerization process, even if an increase in the ionic conductivity in samples containing fibers leads to the hypothesis that samples with fibers are less consolidated, or that fiber dissolution contributes to the conductivity values. The amorphous fraction was enriched in silicon after HCl attack, but the structure was not completely dissolved, and the presence of an amorphous phase is confirmed (C–S–H gel). Basalt fibers partly dissolved in the alkaline environment, leading to the formation of a C–N–A–S–H gel surrounding the fibers. In contrast, cellulose fiber remained stable in both acidic and alkaline conditions.

## 1. Introduction

Solutions that reduce the CO_2_ emission of the Portland cement industry have been actively sought for decades. The cement industry takes responsibility for ca. 8% total CO_2_ emission, which makes this industry the third largest source of anthropogenic carbon emission [1]. The demand for cement is increasing proportionally with the rise in the human population [2]. Therefore, alternative cementitious binders, produced from utilizing industrial side streams with a lower carbon footprint, are favorable to partially replace Portland cement. Among these alternatives, alkali-activated materials (AAMs) have attracted attention as a promising cementitious binder [3,4]. In addition, the use of by-products from other industries, such as metallurgical slags, in making AAMs, fosters the concept of circular economy and hence a sustainable use of resources.

Steel slags have been intensively studied for their use as both the precursor and aggregates in AAMs. In Refs. [5,6], basic oxygen furnace (BOF) slag was carbonated prior to its use as aggregates in AAMs. This approach is effective to reduce, or even eliminate, the availability of free CaO in the slag, which often causes deleterious damages in hardened mortar. Regarding ladle slag, it showed good ability to be used as a precursor for AAMs [7,8,9], an ettringite-based binder [10,11], while other utilization methods, such as road construction and agriculture fertilizer, was also reported in the literature [12]. On the other hand, desulfurization (De-S) slag showed its potential as a precursor for cementitious materials via alkali activation [6]. The bottleneck of both BOF and De-S slag is their high free CaO content, leading to potential deleterious crystallization stress due to late reaction. Therefore, solutions that can overcome this obstacle and enhance the use of these slags, are of high industrial interest. Similarly to all other cementitious materials, AAMs have brittle behavior, particularly under tension; hence, fibers have been used effectively to enhance the mechanical performance and durability of AAMs [13]. However, the interaction between fibers and the alkali-activated matrix, which can influence the chemical stability of alkali-activated composites, AACs, remains unclear.

It is well known from the literature that the main factors affecting the AAMs properties depend on the utilized aluminosilicate source [14,15,16]. Apart from the aluminosilicate source type and size, the water curing regime, alkali activator, concentration of alkali activator, oven curing temperature and its time, and the alkali activator-to-solid weight ratio also strongly affect the dissolution and polycondensation process. Keeping all these parameters constant in the preparation of composite materials, the nature of the reinforcement, basalt fibers, or cellulose fibers in this work, together with the volume or weight fraction (percent) and their aspect ratio, are accounted as effective parameters during the consolidation process. A large number of different types of fibers has already been investigated from the following slag-based inorganic binders: polyethylene fibers [17,18,19]; polyvinyl alcohol (PVA) fibers [20]; carbon fibers [21]; steel [22,23,24,25] and alkali-resistant glass fibers [26], or other functionalizing components [27]. Since the insertion of the reinforcement is expected to improve the mechanical performance of the matrix, or binder, the majority of the published studies present results that are related to the performance and fracture toughness of these composites. However, there was a lack of published data in the open literature, investigating whether these reinforcements will affect the reaction in AAMs or not. Only recently [17,18,19], some studies are more dedicated to the self-healing properties of alkali-activated slag-based composites, where, in NaOH or Na-silicate activated CaO–(Na_2_O)–Al_2_O_3_–SiO_2_–H_2_O (C–(N)–A–S–H) matrices, CaCO_3_ and some amount of Na_2_CO_3_ were formed in the fractured areas. Concerning steel-reinforced AASC [22], it was proved that high-performance fiber-reinforced composites, with respect to Portland cement ones, can be prepared, owing to the alkali-activated matrix, which has superior adherence to steel fiber. The problem that arises due to the high drying shrinkage of AASC, in the fiber–matrix transition zone, can be eliminated by incorporating pozzolans.

The description of the disordered, chemically complex, multiphase nature of the key strength-giving components of cementitious materials, means that the parameters controlling their structure are the goal of this study. The approach is the investigation of the chemical stability of the matrix in fiber-reinforced slag-based alkali-activated composites, in order to assess any possible effect of the presence of the reinforcement on the chemistry of polycondensation. For this purpose, we chose an organic fiber, i.e., cellulose, and an inorganic fiber, i.e., basalt, showing a different behavior in the alkaline media used to activate the slag fine powders, which are the aluminosilicate precursors. To test the degree of polycondensation, we adopted some direct and indirect tests. The evaluation of the chemical behavior of the slag was performed by means of basic attack in NaOH, to evaluate the Si and Al availability from the slag in the typical alkaline environment of alkali-activated materials. Successively, the behavior of alkali-activated composites was studied in water (integrity test, normed leaching test, pH and ionic conductivity), and acids (leaching in acetic acid and HCl attack). These tests were chosen to evaluate the ions mobility, in terms of heavy metal immobilization efficiency and the mobility of matrix elements, such as Si and Al. Diffractometric analysis was a direct test for observing the modification of both crystalline phases (appearance and disappearance) and the amorphous fraction that is always present in the slag, as well as in AACs.

## 2. Materials and Methods

### 2.1. Raw Materials

The following two different slags were used in this research:

(1) Basic oxygen furnace (BOF), coming from SSAB Europe Oy, in Finland. Basic oxygen steelmaking is a method of primary steelmaking in which carbon-rich molten pig iron is converted into steel. Blowing oxygen through the molten pig iron lowers the carbon content of the alloy and changes it into low-carbon steel. The process is known as ”basic” because fluxes of burnt lime or dolomite, chemically classified as bases, are added to promote the removal of impurities. This procedure generates BOF slag. To obtain carbonated BOF, c-BOF, pristine slag was exposed to CO_2_ flow gas in the carbonation chamber (5% CO_2_ concentration, 60% RH, temperature of 23 °C) for 48 h. The calcium oxide content in BOF slag reacts with CO_2_ gas. This reaction leads to the formation of crystalline calcite, CaCO_3_ [28].

(2) Desulfurization slag, De-S, coming from SSAB Europe Oy, in Finland. During steelmaking, a desulfurizer, composed of lime (CaO, ~90%) and calcium fluoride (CaF_2_, ∼10%), is used in the secondary refining process. A layer of slag floating on the liquid iron forms and sulfur is removed from the liquid iron by a slag–liquid metal reaction, with the following reaction:

Ca^2+^_(slag)_ + S^2−^_(iron)_ → CaS _(slag)_

The resulting slag, called desulfurization slag, is primarily composed of excessive lime, entrapped iron, the desulfurization products (CaS and Al_2_O_3_) plus residual fluorides. Although this kind of slag normally contains a considerable amount of iron and iron oxides, it is not often recycled in steel manufacturing because of the high sulfur content.

In Table 1 the chemical composition of the two used slags, obtained by XRF (Bruker AXS S4 Pioneer) are reported. Both the slags contain significant amounts of Ca and Fe, and around 13–17% of SiO_2_ + Al_2_O_3_. The table also shows the loss in ignition, L.O.I, at 950 °C.

**Figure 1:**
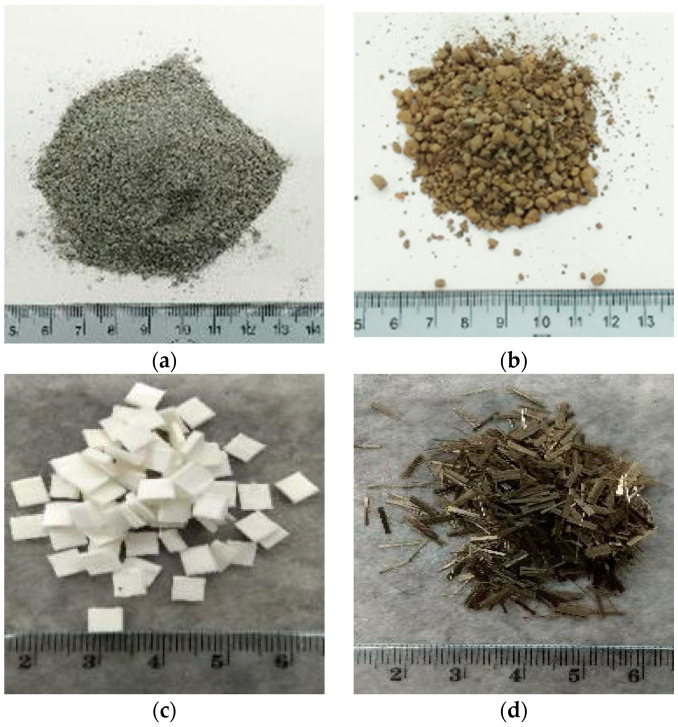
Appearance of raw materials including (**a**) De-S slag, (**b**) carbonated BOF slag, (**c**) cellulose and (**d**) basalt fibers.

In Table 2 the physical characteristics of the two fibers used in this work, i.e., cellulose and basalt fibers, are reported. As expected, basalt fibers have higher density and rigidity with respect to cellulose. Figure 1 depicts the appearance of raw materials and fibers used to realize alkali-activated materials.

**Figure 2:**
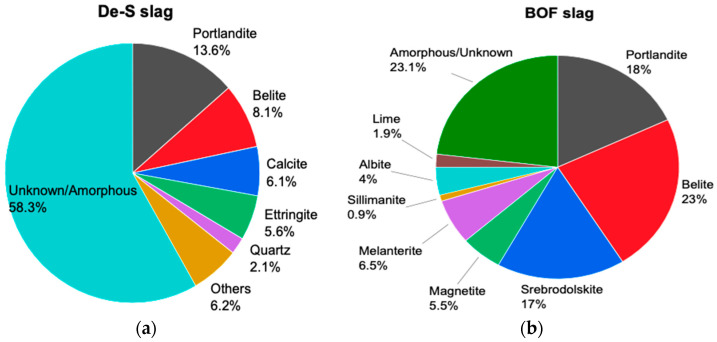
Mineralogy of (**a**) De-S slag and (**b**) BOF slag determined by QXRD.

The mineral composition and quantitative XRD (QXRD) analysis of the two slags were carried out using a Rigaku SmartLab 9 kW with Co-Kα radiation (Kα1 = 1.78892 Å; Kα2 = 1.79278 Å; Kα1/Kα2 = 0.5). The measurements were conducted at a scan rate of 3°/min in the range 5°–90° (2θ) and 0.02°/step. Zincite, ZnO, was used as an internal standard (ca. 10 wt.%) to determine the fraction of amorphous content in the samples. The phase assignment was conducted with PDXL V.2 (Rigaku, Japan) using the PDF-4+ 2020 database. Mineralogical analysis (Figure 2) of the desulfurization and carbonated slags shows, for the first one, the presence of ettringite confirming the presence of sulfur. In BOF slag, srebrodolskite (Ca_2_Fe_2_O_5_), portlandite (Ca(OH)_2_) and lime (CaO) confirm the high content of Ca and the addition during the steelmaking process of lime and dolomite. An important difference between the two slags is the amorphous content, indeed in BOF it is 23% while in De-S it is 58%.

### 2.2. Preparation of Alkali-Activated Mixtures

De-S slag was used as a precursor to form the binder matrix in this work. Prior to its use, the slag was sieved to achieve a maximum particle size less than 0.1 mm. On the other hand, carbonated BOF slag was used as aggregates in mix composition; hence, the as-received slag was sieved to have a grain size smaller than 0.5 mm prior to its carbonation. The alkaline activator consisted of Na_2_SiO_3_ and NaOH with a silica modulus of 2.5 mol/mol, while the concentration of NaOH was 8M, as optimized in previous study [6]. The alkali solution was prepared at least 1 day to completely cool down the solution prior to its use in making samples; this helps eliminate the undesirable effects of heat generation on hardened state properties as well as attaining a more homogenous solution.

Table 3 shows the mix recipes of mixtures in this study. The mixing procedure, detailed in Ref. [6], is as follows. The dry powders of De-S slag and BOF aggregates were mixed together for 2 min. The alkaline activator was added to the dry mixture and stirred for ca. 2 min. After attaining a homogenous mortar, fibers, prepared according to M2 or M3 as in Table 3, were gradually added 4 wt.% (in the mass of the binder) to the fresh paste and mixed for another 3 min. The mixture was cast in oiled plastic molds and sealed with plastic bags for 24 h. Specimens were demolded after 1 day of curing and sealed in plastic bags for further analyses (detailed in Section 2.3).

### 2.3. Characterization and Reactivity of Slags and Alkali-Activated Materials

The chemical stability and degree of polycondensation of the samples in aqueous environment was analyzed by integrity test [29]. The test requires that a specimen of 3–4 g is immersed in acetone for at least 2 h in order to eliminate the humidity and pores solution. Then the sample was weighed and immersed in distilled water for 24 h (S/L ratio wt.% 1:100). After this soaking time, the piece was immersed in a beaker with acetone for 2 h, then naturally dried and weighted in order to evaluate the mass loss.

To assess the hazardous or not hazardous nature of the raw materials and associated AAC, as well as their behavior towards the rain, leaching tests were performed. These tests were carried out in the following three different environments: basic, neutral and acid pH. In particular, the following:

- Reactivity test in NaOH on as-received slags.

The test was performed on the as-received slags in order to quantify the amount of Al^+3^ and Si^+4^ ions that are released into the solution during the dissolution step of the geopolymerization, simulating the chemical attack of the alkaline environment during activation. In order to evaluate the reactivity in highly concentrated NaOH of the three slags, and their suitability to be used as starting aluminosilicate powders for the production of alkali-activated binder, a dissolution test in NaOH was performed [30]. Slag was immersed in NaOH, 8M, solution with a solid/liquid weight ratio = 100 and stirred constantly for 5 h in a flask bathed at 80 ± 2 °C. The individuation of the crystalline phases that were dissolved during the alkaline attack was conducted by collecting XRD before and after the treatment (for XRD details, see next section).

- Leaching test in distilled water on as-received slags and AAM + AACs.

In order to evaluate the leachability of the heavy metals from either the raw materials/slags and the alkali-activated materials, a leaching test in distilled water was performed according to the European standard UNI EN 12457-2. This experimental procedure had the final goal to observe if the basalt or cellulose fibers used as reinforcement in the AACs affected the release of heavy metals from the matrix, assuming that the heavy metals contained in the basalt fibers were released at a very minor extent due to their more stable microstructure.

Ten grams of solid shreds of specimens were sieved under 2 mm and immersed in distilled water with a solid/liquid ratio wt.% of 1:10 into a Teflon^®^ bottle for 24 h in stirring conditions. Then, the mixture was filtered and the liquid fraction was acidified with HNO_3_ to pH = 2. The eluate was analyzed by ICP/OES (Varian Liberty AX) to estimate the concentrations of heavy metals released [29,31].

The measurements of pH and ionic conductivity were performed on the leachate solution on a 1:10 solid-to-water weight ratio to assess the chemical stability of the slags in terms of degree of consolidation of the alkali-activated materials. These measurements are directly dependent on the number of ions released by the AAM samples, hence they can indicate the extent of polycondensation. The leachate solution’s pH (pH sensor Hamilton type Liq.glass SL) and ionic conductivity (OAKTON Eutech Instruments COND6/TDS 6) were determined during leaching test for the following different times: 0, 5, 15, 30, 60, 120, 240, 480, 1440 min [29,31,32].

- Leaching test in acetic acid on as-received slags and AAM + AACs.

The acetic acid test was performed to assess the behavior of the alkali-activated materials under the leaching action of meteoric water and acid rains, and to determine the amount of the contaminants after their contact. This test was chosen based on common testing programs reported in the literature for geopolymers and AAMs [33,34]. This analysis [35,36], according to European Directive 91/271/CEE concerning urban wastewater treatment, was also performed on the as-received slags in order to estimate their leaching behavior by comparing the results with that of alkali-activated composites.

For the procedure, a sample of 10 g of samples was immersed in distilled water (solid/liquid ratio wt.% 1:16) and stirred constantly for 24 h on a magnetic stirrer at room temperature. The grain size of the sample was less than 9.5 mm. The solution was maintained at pH < 5 (pH-meter OAKTON Eutech pH 5/6 and Ions 6 and a pH electrode Polyplast—Hamilton 0–14 pH) adding acetic acid solution 0.5 N. The pH correction was made at least every 15 min during the first hour and hourly during the following period. After the first 24 h of the test, if the pH was higher than 5, the pH monitoring continued for a further 4 h. The final solution was then filtered to separate solid from liquid and the liquid phase was increased to 20 times the sample weight by adding distilled water. The liquid fraction was acidified at pH = 2 with concentrated nitric acid in order to evaluate the release of heavy metals with ICP/OES (Varian Liberty AX).

- Test in hydrochloric acid.

In order to provoke the dissolution of the main products of the geopolymerization process, the acid attack in HCl was performed. This experimental procedure quantified the amount of unreacted raw materials as insoluble fraction and the raw materials that had been transformed, after alkaline activation, to a stable and compact network as soluble fraction. Moreover, these results were a reference to estimate the role of fibers and their influence on a matrix of alkali-activated material. The procedure adopted in this work finds references already reported in the literature [36]. One gram of slags, or of AACs, (grain size under 1 mm) was immersed in 250 mL of HCl solution (1:20 by weight) in stirring conditions for 3 h at room temperature. The mixture was filtered and the insoluble fraction was washed with distilled water to rise pH = 7, it was dried at 105 °C in an oven for two hours and then weighed; the soluble fraction in the liquid was acidified with HNO_3_ to pH = 2 and it was analyzed by ICP/AES (Varian Liberty AX).

The insoluble fraction obtained by acid attack was analyzed by XRD. Mineralogical characterization was performed to compare the crystalline phases present in the as-received slags and basalt fibers and alkali-activated material after the geopolymerization process. This test was carried out by a powder diffractometer (PW 3710, Philips) with Cu Kα radiation in the 5–70° 2θ range on powder samples of 20–30 μm in size. This technique was also used to analyze samples before and after all the chemical attacks performed, but in order to obtain quantitative results.

TGA/DTG analyses were conducted with a DSC 2500 by TA instrument (USA) with a powdered sample’s mass of ca. 20–30 mg. Alumina crucibles were used in experiments, and temperature ranged from 25 °C to 1000 °C with a heating rate of 10 °C/min in a nitrogen atmosphere with a flow rate of 20–50 mL/min.

The microstructure and fiber–matrix interface of samples were observed using scanning electron microscopy (SEM) with a Zeiss Ultra Plus (Germany). The accelerating voltage was set to 15 kV, while backscattered electron (BSE) was used at a working distance of 8–12 mm. The samples were cast into low-viscosity epoxy resin by vacuum-impregnating. After the hardening of the resin, samples were polished with diamond discs (to 1 μm size) at 150 rpm using ethanol. The samples were coated with carbon. Chemical compositions were determined using an X-Max EDS detector (Oxford Instrument, the UK). Regarding the observation for fibre–matrix interface, samples were collected from freshly fracture surfaces without casting into epoxy and polishing.

## 3. Results and Discussion

### 3.1. Carbonated and Desulfurization Slags Characterization

In order to determine the potentially reactive fraction that is present in the slags, as a precursor in the production of alkali-activated materials, the alkaline attack was performed. The reactive Si/Al mass ratio, calculated from the number of Si and Al ions released into the alkaline solutions, provided information about the suitability of the raw materials to form the alkali-activated materials. The concentrations of these elements are reported in Table 4. The results showed that both the slags present very low releases of Si and Al, with respect to the reference material metakaolin (Si = 194 ppm, Al = 144 ppm, and Si/Al = 1.35, see Supplementary Materials). The c-BOF slag presented a higher number of Si and Al ions in the leachate, and a more proper ratio with respect to the De-S slag. For both the slags, the Si content in the solutions is higher with respect to Al, according to both the chemical compositions (Table 1), in comparison to metakaolin.

The Si/Al mass ratio is correlated with the reticulation degree of the alkali-activated materials; in fact, when the Si/Al mass ratio is below the value of three, the final materials are characterized by a 3D rigid network, which would be a proper matrix for a cement, mortar, or concrete [31,37]. Fletcher et al. [37] observed that the raw materials that are characterized by a Si/Al mass ratio below the value two, do not show the typical properties of geopolymers. The previous results confirm the findings of the authors, for the significance of the relation between the Si/Al mass ratio and qualifying the reactivity of raw materials that are suitable in the realization of AACs.

Furthermore, heavy metals and amphoteric elements, such as Sb, Se, and As, are below the detection limits, and Mo was only slightly released (Table 4).

**Figure 3:**
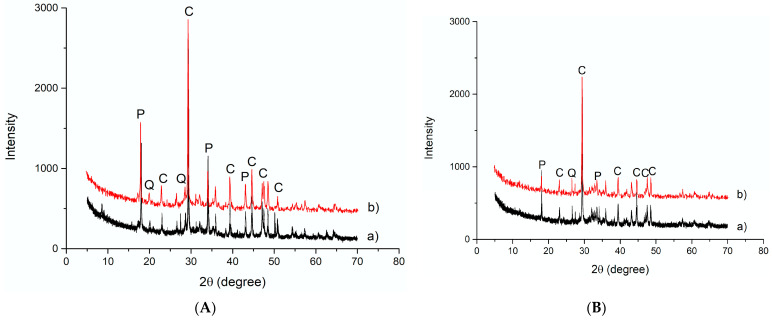
XRD patterns collected on (**A**) carbonated slag and (**B**) desulfurization slag before (a) and after (b) NaOH 8M test (C = calcite; P = portlandite; Q = quartz).

Mineralogical analysis was performed to evaluate if the crystalline phases in the starting slags changed or disappeared after the alkaline attack (Figure 3a,b). Carbonated slag (Figure 3A) was characterized by Q-quartz (SiO_2_), C-calcite (CaCO_3_), and P-portlandite (Ca(OH)_2_). After immersion in the NaOH 8M solution, no significant variation was observed (Figure 3a). For desulfurization slag, a flattening of the band between 25 and 40 2θ, corresponding to the aluminosilicate amorphous phase, is visible, confirming the higher reactivity of the glassy fraction in an alkaline environment (Figure 3B). The higher reactivity of the amorphous phase in the NaOH 8M solution was previously evidenced by the authors for incinerator bottom ash, showing a significant decrease in the amorphous hump after the treatment [38].

**Figure 4:**
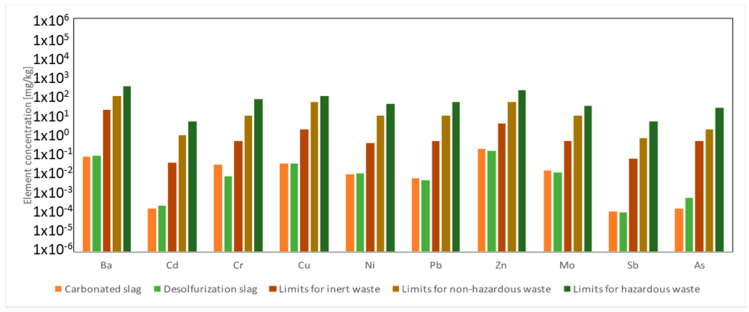
Heavy metals (mg/L) in the slags carbonated and desulfurization eluates after leaching test in water (EN 12457-2) and comparison with the limits defined in 2003/33/EC for inert, non-hazardous and hazardous wastes (due to variability of the raw materials and the analytical process, the error associated with the measure is 30%).

In order to investigate the eventual hazardous nature of carbonated BOF and De-S slags, the leaching test, according to the European standard EN 12457-2, was carried out. The concentrations of heavy metals, such as Pb, Cd, and As, into a leachate (Figure 4), were compared to the limit values that were included in the council decision 2003/33/EC prescribed to place not dangerous waste in landfill (law limit in water).

The concentrations of heavy metals found in the eluate were lower than the law limit values, confirming that these slags are not dangerous raw materials and can be safely used for the alkali-activated materials.

**Figure 5:**
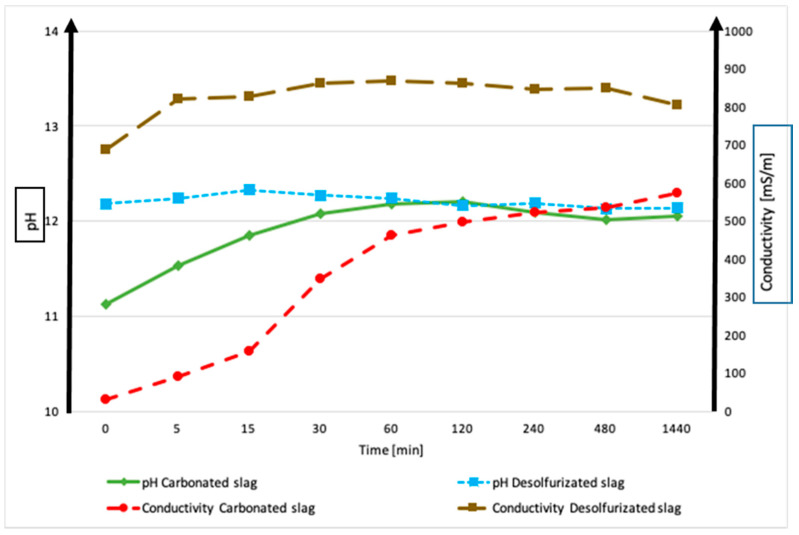
pH and conductivity of carbonated and desulfurization slags. The error associated with these measurements is approx. 2% for pH and 8% for conductivity caused by sample variability.

During the leaching test (EN 12457-2), the pH and ionic conductivity of the c-BOF and De-S slags were measured at the following different times: 0, 5, 15, 30, 60, 120, 240, 480, and 1440 min, after immersion in water (Figure 5). These measurements have been useful to analyze the chemical stability of raw materials that are in contact with water, as already reported in the previous paper [9,29]. Starting from pH = 7, all the slags showed an increase until the constant pH value around 12, during the first 24 h; instead, their conductivity grew rapidly in the first hour, to reach constant values. C-BOF slag’s conductivity increased continuously, reaching the value of 600 mS/m. Although De-S slag showed the highest conductivity values, it was the most stable of the two slags because it immediately released the largest number of ions in the aqueous environment, yet rapidly reached a constant value (800 mS/m) (see the plateau in Figure 5). On the contrary, carbonated slag was characterized by a continuously increasing trend in the release of ions during the 24 h of the test. This behavior shows that the leaching of ions is progressive for c-BOF, while for De-S, after the first minutes, the slag is stable without the leaching of ions. The progressive release of ions into NaOH solution, reached the higher values of the heavy metals content (see Table 4) for c-BOF.

**Figure 6:**
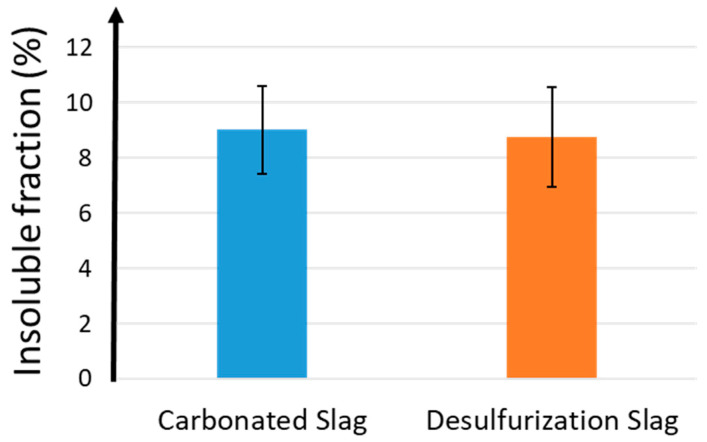
Insoluble fraction after HCl test of c-BOF and De-S slags.

The carbonated and desulfurization slags presented low percentages of insoluble residual fraction in HCl. The high amount of calcium oxide, 54 and 55 wt.%, respectively, justifies the high solubility in hydrochloric acid (Figure 6). The high solubility in HCl is related to both the dissolution of the amorphous gel, formed during alkali activation, and to the presence of crystalline phases that are soluble in HCl, such as calcite. The contribution of the soluble crystalline phases leads to a lower amount of insoluble fraction when compared to metakaolin, which shows 36% (see Supplementary Materials).

The soluble fractions of all the raw materials were analyzed by ICP/AES, in order to obtain information about the presence of elements that dissolved from the solid structure of the materials. For such a reason, Table 5 does not show the law limit for these leachate values, since this is a structural leaching and not a chemical leaching test. All the metals show low values of release, except chromium, because its leaching increases after carbonation, due to pH reduction. 

**Figure 7:**
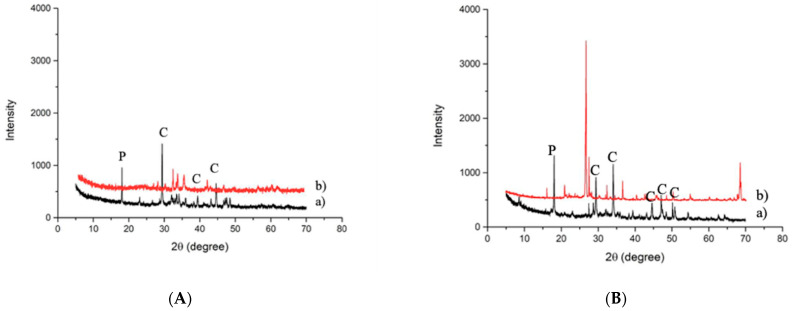
XRD patterns collected on (**A**) c-BOF slag and (**B**) De-S slag before (a) and after HCl test 1:20 (b) (C = calcite, P = portlandite).

The insoluble residual fraction of all the slags was analyzed by XRD (Figure 7), in order to observe the modifications of the crystalline phases after the acid attack. Figure 7 shows the dissolution of calcite (CaCO_3_) and portlandite (Ca(OH)_2_) after the HCl test 1:20 for both the slags.

The acetic acid test was performed in order to assess the behavior and stability of the investigated AACs to acid rains. This analysis was also carried out on slags, to evaluate the role of geopolymerization, in terms of the chemical stability of the corresponding ACCs. Thus, the goal of the acetic acid attack was to compare the metals that were released into distilled water and the corresponding amount released after the contact with the acid solution (Table 5), and the possible modification of the crystalline phases after the test.

The release of heavy metals was compared with the law limits that are contained in the European Directive 1991/271/CEE concerning urban waste water treatment. Table 5 shows that the presence of each metal in the liquid fraction is below the law limits, meaning that the leachate that was left free after the contact between the acid rains and materials was not dangerous for urban water.

### 3.2. AAM and AAC Characterization

**Figure 8:**
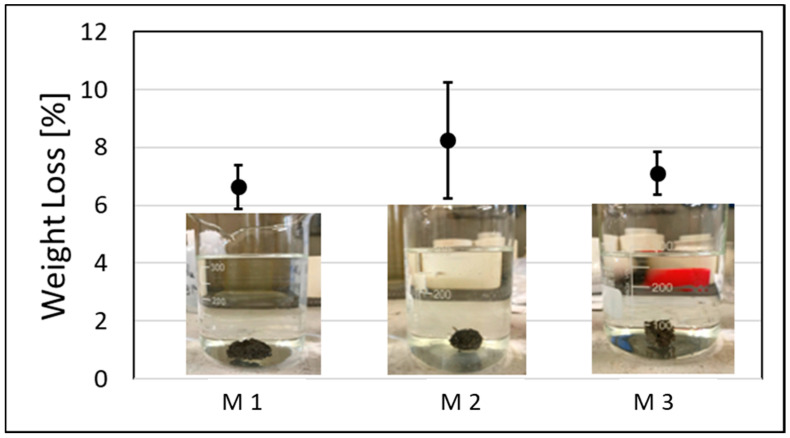
Samples M1, M2, M3 after the integrity test and their weight loss [%].

The structural and chemical stability of the AAM (M1) and AACs (M2 and M3) were analyzed by an integrity test, as described in Section 2.3. After immersion in the distilled water for 24 h, all the specimens were stable, recording a weight loss of 6–8 wt.%, confirming the occurrence of the geopolymerization reaction (Figure 8).

The integrity test in water was performed to assess the structural stability of the samples, while the quantitative evaluation of the chemical stability was measured by the weight loss (Figure 8). The weight loss range was between 6.5 to 8.2%, but the wide variability of the samples generated an error of approx. ±2%. The reinforced samples, with basalt fibers (specimen M2), seems to have higher weight loss with respect to the reference AAM (specimen M1) that did not contain any fibers, but, due to the sample variability, such difference cannot be considered significant (Figure 8).

**Figure 9:**
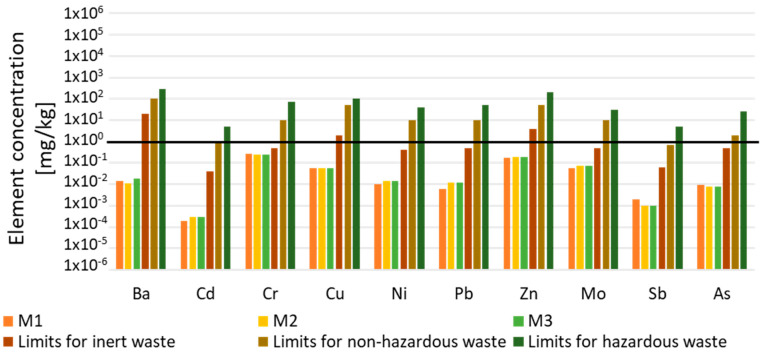
Heavy metals (mg/L) in M1, M2 and M3 AACs after the leaching test in water (EN 12457-2) and comparison with the limits defined in 2003/33/EC for inert, non-hazardous and hazardous wastes (the error associated with the measure is 30%).

The capability of the AAMs materials to immobilize heavy metals into their matrix, was evaluated by the leaching test that is prescribed in the European standard EN 12457-2. The content of heavy metals in the eluate of M1, M2, and M3 was compared to the limits that are prescribed to dispose of non-dangerous waste in landfill (council decision 2003/33/EC) (Figure 9). All the compositions present leaching values below the law limit for landfill for not dangerous waste, confirming that the stability that is reached with the occurrence of geopolymerization is higher than the enhancing of ions mobility, due to the alkaline pH that is typical of alkali-activated materials.

The presence of the fibers in the specimens did not influence the release of the heavy metals in the eluate; the values found of M2 and M3 were very similar to those of M1. A slight increase in the metals release in the reinforced composites is evident for Cd, Ni, and Pb. The presence of the fibers does not affect the ions’ release, which is in line with the absence of influence on the cold consolidation. Other authors stated that harmful water environments (similar to the alkaline environment that is typical of geopolymers) did not weaken the effect of the composite fiber in the asphalt matrix [39]. An interesting point is the comparison of the release values of the amphoteric elements Mo, Sb, and As, from the as-received slags (Figure 4) and their corresponding AAMs in the distilled water (Figure 9). The results showed the increase in Sb, Mo, and As after the alkaline activation, still remaining below the law limits, as already observed for other kinds of metallurgical slags [9].

**Figure 10:**
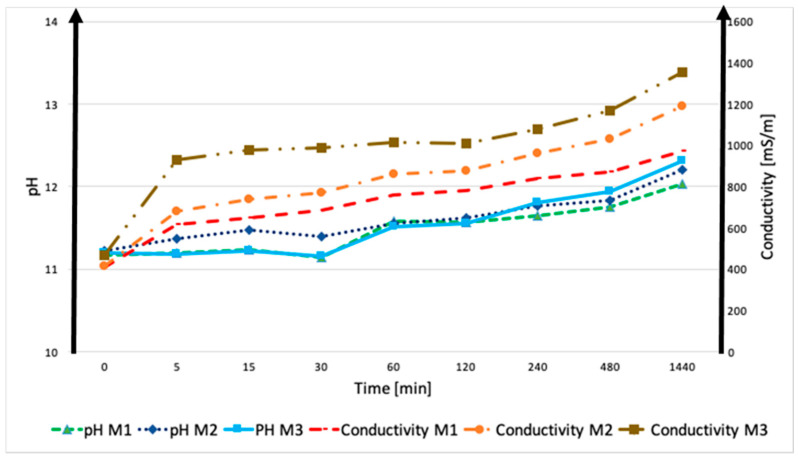
pH and conductivity of the following alkali-activated composites: M1, M2, M3. The error associated to these measurements is approx. 2% for pH and 8% for conductivity caused by sample variability.

During the leaching test, the consolidation performance was investigated by pH and ionic conductivity measurements, which were taken as indicators of the chemical stability of alkaline-activated materials. For all the samples, there was not a significant modification of pH values; M1, M2, and M3 showed a pH range from about 11.2, after a few minutes from immersion, to around 12.2, after 24 h. The fibers that were added to the M1 matrix did not influence the behavior in terms of ions release (Figure 10). For all the three alkali-activated materials, there was a significant increase in the conductivity values during the 24 h, in a more gradual way [31]. In particular, M1 showed lower values of ionic conductivity (from 400 mS/m to 900 mS/m) with respect to M2 and M3, containing basalt and cellulose fibers, respectively. M2 showed values ranging from 400 mS/m to 1200 mS/m, and M3 from 450 mS/m to 1350 mS/m (Figure 10). The presence of the fibers in the AAM matrix did not favor the consolidation of the material, and it was possible to observe, from the conductivity values, the release of a significant number of ions in the aqueous environment for the AACs, probably Na^+^ coming from unreacted activating solutions. This behavior can be related to a lower degree of consolidation of M2 and M3, with respect to M1. These results were confirmed by the weight loss values (Figure 8) and by the ionic conductivity values (Figure 10), while the differences in the release of other metals (Figure 9) are less than 0.1 mg/kg. From the results of the previous analyses, sample M1 (reference specimen without fibers) was characterized by the best properties, in terms of chemical stability, whilst the presence of the fibers enhanced the release of ions. Moreover, M3 showed higher values of conductivity during the first 24 h; the presence of fibers involved no improvements in the properties of the material, probably due to the incomplete dispersion of the fibers within the matrix, as observed by the SEM micrographs.

To investigate the behavior of the AAM (M1) and AACs (M2 and M3) on the attack of acid rains, an acetic acid test was performed. This test was also carried out on the as-received slags for the sake of comparison. The results showed that the raw materials did not release heavy metals on the urban waste water, so they were suitable to realize AACs. The same considerations are valid for the alkali-activated materials. The soluble fractions, the eluates of the acetic acid test, were analyzed by ICP/AES. Table 6 shows that the release of heavy metals was below the limits contained in the council decision 2003/33/EC, concerning urban waste water treatment, so these materials are non-dangerous for the environment.

**Figure 11:**
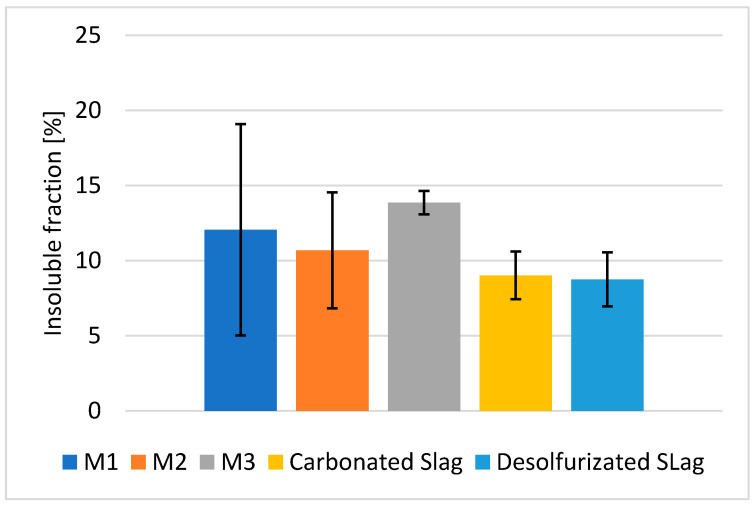
Insoluble fraction after HCl test of M1, M2, M3, c-BOF slag and De-S slag.

The percentage of reaction products that was generated during alkali activation was determined by acid attack with HCl solution, according to the literature [37]. After the acid attack, insoluble and soluble fractions were produced. In particular, the insoluble fraction has to be considered as the part of the consolidated AAM/AAC that had not reacted with the alkaline solutions. From Figure 11, it was possible to observe the insoluble fractions of M1, M2, and M3, which are very similar, especially considering the error bar. M2 was characterized by a lower insoluble phase with respect to M1 and M3, but taking into account the natural compositional variability, no significant differences are recognized. As for all the metallurgical slags, the presence of crystalline soluble phases increases the solubility of the samples, with respect to metakaolin [9]. In the literature [40], the effect of steel fibers on the durability performance of alkali-activated materials were studied, showing that the incorporation of fibers has no significant effect on the acid attack. Fiber-reinforced alkali-activated materials present a mass loss for chemical attacks, but the loss in the Portland materials was approximately twice that recorded for the AAMs.

**Figure 12:**
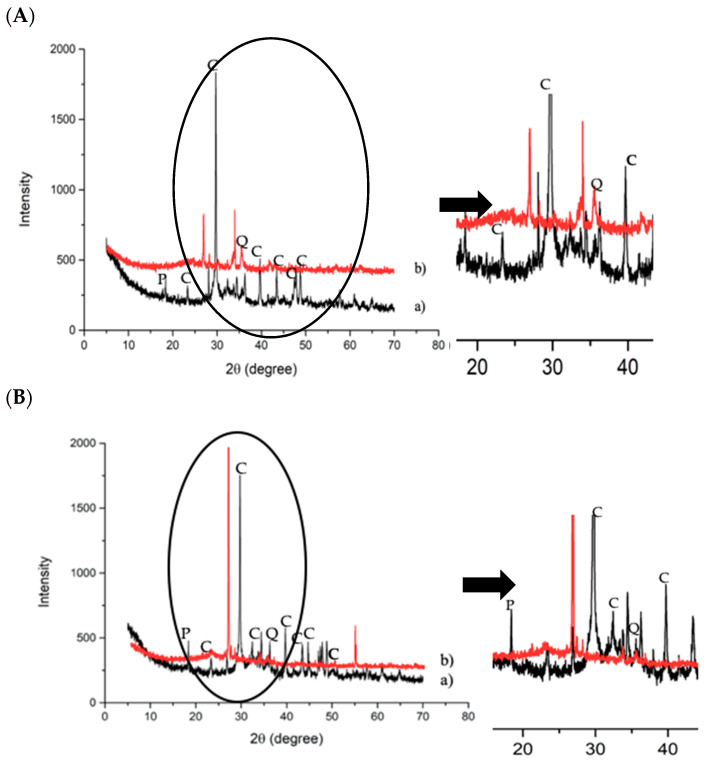

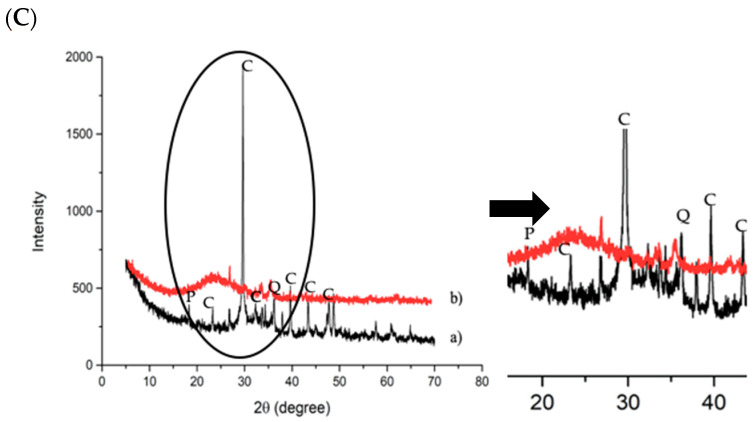
XRD pattern of AAM (a) and AAM after HCl (b): (**A**) M1 (**B**) M2 and (**C**) M3 (Q = quartz; C = calcite; P = portlandite).

The insoluble fraction that was collected after the acid attack was analyzed by XRD, to investigate the crystalline phase changes (Figure 12a–c). All the residues after the HCl test showed the dissolution of calcite and dolomite, which significantly contributed to increasing the amount of soluble fraction. The presence of the quartz crystals after the acid attack confirmed that it was not soluble in hydrochloric acid. The fraction of amorphous content in all the mixes remained stable and comparable among the developed materials after exposure to the acid. All the mixes contained ca. 60–65 wt.% amorphous content after the acid attack, showing good stability of the C–S–H gel in the materials. Note that this relative fraction slightly increased compared to the non-exposing samples, since the dissolution of portlandite and calcite led to the higher proportion of the C–S–H phase.

In all the cases, it should be noted that the broad peak, indicating the presence of the amorphous phase, was not completely dissolved in HCl, but shifted its position towards low two-theta degrees, with respect to the as-prepared AACs, moving from 25–40° 2θ to 20–30° 2θ after the acid attack. This particular behavior can be explained with the modification of the chemical composition of the amorphous gel phase, which becomes richer in silica after the leaching of Al out from the aluminosilicate geopolymer matrix. The Si-rich amorphous glass is typically localized at lower two-theta values (10–35° in 2θ) with respect to the aluminosilicate amorphous phases [41]. This behavior can also explain the increase in the insoluble fraction of AAM and AAC, with respect to the as-received slags after HCl attack, as reported in Figure 11.

**Figure 13:**
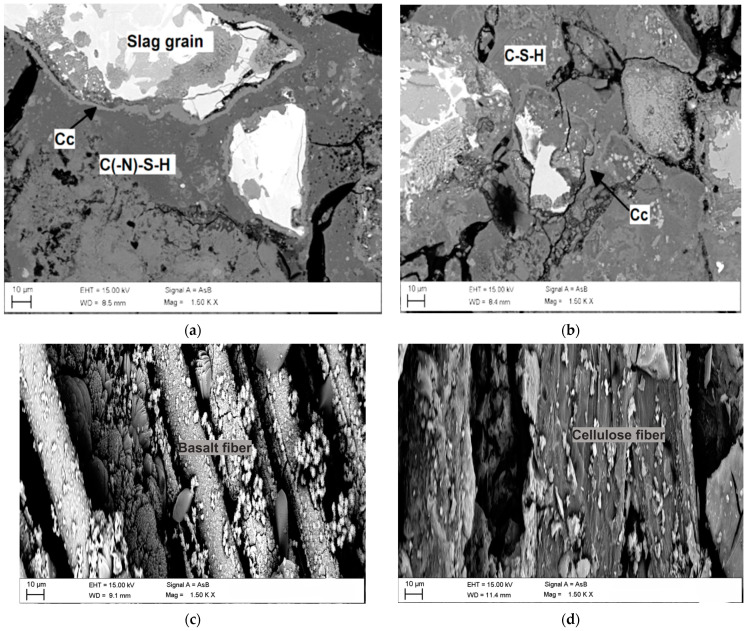
SEM micrograph of M1 AAM before acid attack (**a**), M1 AAM (**b**), M2 AAC (**c**), M3 AAC (**d**), after HCl attack.

Moreover, the insoluble fractions that were obtained by the HCl test were analyzed by scanning electron microscopy (SEM), to investigate the microstructure of AACs before and after the acid attack. In particular, Figure 13a shows M1 AAM, where calcite was formed at the interface surrounding the slag grains, as the result of the carbonation process for BOF slag, which are used as aggregates in the formulation. There was a fraction of unreacted BOF slag in the binder, likely due to the covering of calcite from the carbonation process, preventing further reaction of the slag during alkali activation. The acid attack led to the dissolution of portlandite, and the C–S–H gel remained in the matrix after acid exposure, as visible in Fig 13b. Figure 13c showed the effects of M2 AAC (presence of basalt fibers in the matrix); the basalt fibers seem to dissolve partly, to form some C–N–A–S–H phases. This was likely due to the high alkali environment that can cause the dissolution of basalt fibers, or may also come from the acid attack. This, on one hand, can increase the bonding between basalt fibers and the AAM matrix. On the other hand, the contribution of fibers in delaying crack propagation seems delayed, since the debonding process was less effective. In contrast, the cellulose fibers remained stable in the AAM matrix. Moreover, there was no clear damage observed on the fibers after the acid exposure. This observation can explain the better increase in the compressive and flexural strength of the cellulose fiber-reinforced De-S slag-based AAM compared to that of the basalt fiber-reinforced AAC [6].

**Figure 14:**
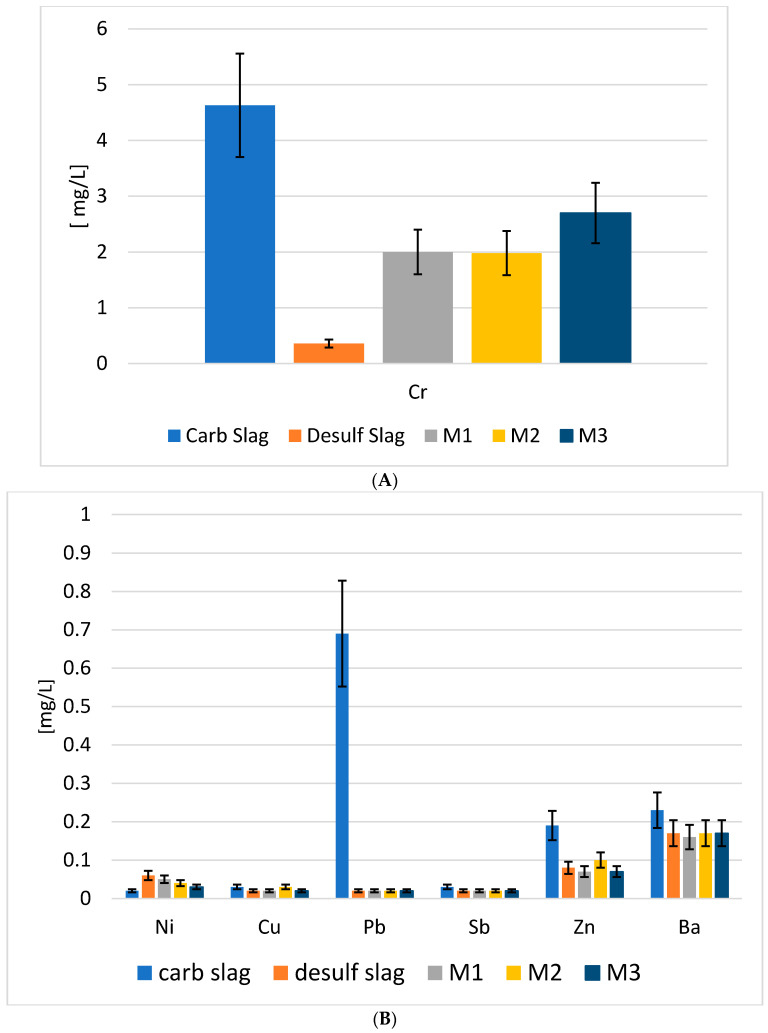
ICP/OES of carbonated slag, desulfurization slag, M1 AAM, M2 AAC and M3 AAC after acid attack in HCl solution (**A**) chromium, (**B**) other metals.

The soluble fraction that was generated by the acid attack of c-BOF and De-S slags, and their AACs were analyzed by ICP/OES, to observe if the release of heavy metals was decreased in the acid environment (Figure 14). The carbonated slag releases a higher content of heavy metals, in particular, Cr, Zn, and Pb, than the desulfurization slag. Instead, the release of As, Cd, and Mo was the same for carbonated and desulfurization slags. The geopolymeric network reduced the release of all metals with respect to the corresponding slags; as an example, Pb, which reduced its values from 0.7 mg/L to a value below 0.1 mg/L.

**Figure 15:**
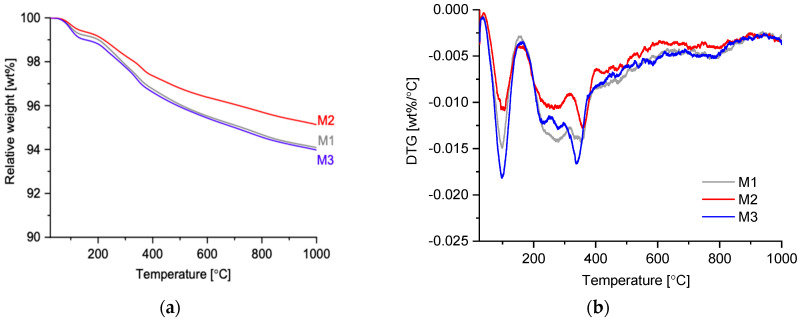
TG (**a**) and DTG (**b**) of residual materials after exposure to HCl.

The residual M1, M2, and M3 after exposure to HCl, were subjected to thermal analysis (TG/DTG) (Figure 15). The mass loss was very small, where M1 and M3 are identical, with a weight loss around 6% and M2 around 5%; this confirms the SEM data showing that the cellulose fibers remained stable in the AAM matrix. While M2 exhibited the least mass loss among the three mixes. The weight losses were attributed to the dehydration (ca. T < 200 °C) and the dehydroxylation, which was typical for geopolymeric matrices for T = 200–400 °C. In addition, there was a very slight thermal event at ca. 800 °C, which was attributed to the decarbonation of leftover calcite. This indicates that likely not all calcite has dissolved after the acid exposure.

## 4. Conclusions

Metallurgical slags were used as precursors as well as aggregates in the alkali-activation process, to obtain chemically stable solid panels. Two different kinds of fibers, basalt and cellulose, were used as reinforcement, in order to improve the mechanical properties. Their chemical performance, and disruption of the structural units in water and acidic media (acetic and hydrochloric acids), was checked via chemical analysis of the leached ions. The presence of fibers does not favor nor hinder the geopolymerization process, even if an increase in the ionic conductivity in samples containing fibers leads to the hypothesis that samples with fibers are less consolidated, or that the partial dissolution of fibers can contribute to conductivity values. The aluminosilicate gel and carbonate fraction are attacked by HCl, with a corresponding enrichment of the amorphous fraction in silicon and a decrease in aluminum, but the structure is not totally dissolved and the presence of an amorphous phase is confirmed; this ensures that the incorporation of fibers has no significant effect on the acid attack and that the cold consolidation occurs, forming a stable matrix.

The microstructure of the binder, M1, indicates that a certain fraction of the C–S–H gel remains stable after the acid attack, while calcite and portlandite were dissolved, as observed in the XRD and SEM characterizations. Basalt fibers partly dissolved in the high pH, caused by the alkali activators, leading to the formation of a C–N–A–S–H gel surrounding the fibers. In contrast, the cellulose fiber remained stable after the acid attack, as well as in the high alkaline environment.

## Figures and Tables

**Table 1:** Overall chemical composition of slag (wt.%) (X-ray fluorescence).

Oxides [wt.%]	c-BOF Slag	St.dev	De-S Slag	St.dev
SiO_2_	13.06	0.12	16.58	0.18
Al_2_O_3_	1.27	0.05	2.4	0.07
Fe_2_O_3_	22.72	0.11	17.57	0.05
CaO	57.96	0.09	60.44	0.13
MgO	2.02	0.07	1.75	0.16
SO_3_	0.21	0.05	5.46	0.28
LOI	2.76		9.49	

**Table 2:** Physical properties of cellulose and basalt fiber used in the present investigation *.

Type	Basalt Fiber	Cellulose Fiber
Length (mm)	6	3
Diameter (m)	17	15
Young’s modulus (GPa)	100	8.5
Tensile strength (MPa)	4500	750
Density (g/cm^3^)	2.63	1.10

* Technical data were retrieved from fiber suppliers.

**Table 3:** Mix recipes of plain mortar (M1) and fiber-reinforced mixtures (M2 and M3).

Specimen ID	De-S Slag (g)	BOF Aggregate (g)	Alkali/Binder Ratio	Fibre (wt%)	Silica Modulus (mol/mol)	NaOH (M)
M1	100	300	1	-	2.5	8
M2	100	300	1	4% basalt fibre	2.5	8
M3	100	300	1	4% cellulose fibre	2.5	8

**Table 4:** ICP-OES determination of the soluble Si and Al atoms, heavy metals and amphoteric elements in the leachate after the NaOH 8M solution test at 80 °C (the error associated with the measure is 30%), DL = detection limit.

Sample/Element (mg/L)	c-BOF Slag	De-S Slag
Al	19	1.7
Si	68	15
Si/Al	3.55	8.67
Cr	0.17	0.02
Ni	0.13	0.09
Cu	0.20	0.06
Zn	0.06	0.03
As	<DL	<DL
Se	<DL	<DL
Mo	0.13	0.32
Cd	<DL	<DL
Sb	<DL	<DL
Ba	0.01	0.05
Pb	<DL	<DL

**Table 5:** Heavy metals (mg/L) in c-BOF and De-S slags’ eluates in acetic acid solution 0.5 N and in HCl solution 1:20 (the error associated with the measure e is 30%).

Heavy Metals (mg/L)	Carbonated Slag		Desulfurization Slag		Law Limits
	Acetic Acid	HCl Acid	Acetic Acid	HCl Test	Acetic Acid
Ba	0.06	0.23	0.21	0.17	20
Cd	<0.02	<0.02	<0.02	<0.02	0.02
Cr	<0.02	4.63	<0.02	0.36	2
Cu	<0.02	0.03	<0.02	0.02	0.1
Ni	<0.02	0.02	0.02	0.06	2
Pb	<0.02	0.69	<0.02	<0.02	0.2
Zn	0.06	0.19	0.05	0.11	0.5
Mo	<0.02	0.02	<0.02	<0.02	-
Sb	<0.02	0.03	<0.02	<0.02	-
As	<0.04	<0.04	<0.04	<0.04	0.5

**Table 6:** Heavy metals (mg)L) in the AACs M1, M2, M3 eluates after the leaching test in acetic acid solution 0.5 N and in HCl solution 1:20 (the error associated with the measure is 30%).

Heavy Metals (mg/L)	M1		M2		M3		Law Limits
	Acetic Acid	HCl Acid	Acetic Acid	HCl Test	Acetic Acid	HCl Test	Acetic Acid
Ba	0.06	0.16	0.08	0.17	0.06	0.17	20
Cd	<0.02	<0.02	<0.02	<0.02	<0.02	<0.02	0.02
Cr	<0.02	2	<0.02	1.98	0.05	2.7	2
Cu	<0.02	0.02	<0.02	0.03	<0.02	0.02	0.1
Ni	<0.02	0.05	<0.02	0.04	<0.02	0.03	2
Pb	<0.02	0.02	<0.02	0.02	<0.02	<0.02	0.2
Zn	0.03	0.07	0.03	0.1	0.04	0.07	0.5
Mo	0.02	<0.02	0.02	<0.02	0.02	<0.02	-
Sb	<0.02	0.02	<0.02	0.02	<0.02	0.02	-
As	<0.04	<0.04	<0.04	<0.04	<0.04	<0.04	0.5

## Data Availability

The data that support the findings of this study are available from the corresponding author upon reasonable request.

## Supplementary Materials

The following are available online at https://www.mdpi.com/article/10.3390/polym13162664/s1, Table S1: Metakaolin and metakaolin geopolymers behaviour in HCl (1:20 by weight), Table S2: metakaolin behaviour in NaOH solution.
