# Supplementary material for: The Effect of Fibrous Reinforcement on the Polycondensation Degree of Slag-Based Alkali Activated Composites
Source: Polymers (Basel). 2021 Aug 10;13(16):2664. doi: 10.3390/polym13162664 (PMC8399163; doi:10.3390/polym13162664)
Supplement: Supplementary file 1 [file polymers-13-02664-s001.zip › polymers-1308270-supplementary.pdf]

polymers

Supplementary materials

The Effect of Fibrous Reinforcement on the Polycondensation Degree of Slag-Based Alkali Activated Composites

Isabella Lancellotti[1,*], Federica Piccolo [1], Hoang Nguyen[2], Mohammad Mastali[2], Mohammad Alzeer[2], Mirja Illikainen[2], Cristina Leonelli[1]

Table S1. Metakaolin and metakaolin geopolymers behaviour in HCl (1:20 by weight) .

	Metakaolin	geoMK 28 days	geoMK 180days
Soluble fraction	23.74%	64.44%	65.8%
Insoluble fraction	76.26%	35.56%	34.2%

Table S2. metakaolin behaviour in NaOH solution.

SAMPLE (µg/ml)	Al	Si	Fe	Si/Al
Metakaolin	144	194	2,9	1,347
